# Monitoring of Current Cancer Therapy by Positron Emission Tomography and Possible Role of Radiomics Assessment

**DOI:** 10.3390/ijms23169394

**Published:** 2022-08-20

**Authors:** Noboru Oriuchi, Hideki Endoh, Kyoichi Kaira

**Affiliations:** 1 Advanced Clinical Research Center, Fukushima Global Medical Science Center, Fukushima Medical University, Fukushima 960-1295, Japan; 2 Department of Nuclear Medicine, Fukushima Medical University Hospital, Fukushima 960-1295, Japan; 3 Department of Thoracic Surgery, Saku Central Hospital Advanced Care Center, Nagano 385-0051, Japan; 4 Department of Respiratory Medicine, Comprehensive Cancer Center, International Medical Center, Saitama Medical University, Saitama 350-1298, Japan

**Keywords:** tumor microenvironment, immunotherapy, FDG-PET, tumor heterogeneity, metabolism, immune-checkpoint inhibitors artificial intelligence, machine learning, radiomics

## Abstract

Evaluation of cancer therapy with imaging is crucial as a surrogate marker of effectiveness and survival. The unique response patterns to therapy with immune-checkpoint inhibitors have facilitated the revision of response evaluation criteria using FDG-PET, because the immune response recalls reactive cells such as activated T-cells and macrophages, which show increased glucose metabolism and apparent progression on morphological imaging. Cellular metabolism and function are critical determinants of the viability of active cells in the tumor microenvironment, which would be novel targets of therapies, such as tumor immunity, metabolism, and genetic mutation. Considering tumor heterogeneity and variation in therapy response specific to the mechanisms of therapy, appropriate response evaluation is required. Radiomics approaches, which combine objective image features with a machine learning algorithm as well as pathologic and genetic data, have remarkably progressed over the past decade, and PET radiomics has increased quality and reliability based on the prosperous publications and standardization initiatives. PET and multimodal imaging will play a definitive role in personalized therapeutic strategies by the precise monitoring in future cancer therapy.

## 1. Introduction

Radiological images have become crucial in clinical practice, for diagnosis and therapy monitoring of cancer patients. Over the past few decades, objective of imaging has progressed from morphology to function and metabolism, as well as from planar to three-dimensional imaging. Progress in computational method and artificial intelligence (AI) has extracted quantitative features from medical images to explore minable data for correlation between these features of tumor as well as surrounding tissues and clinical outcomes. Positron emission tomography (PET) and single-photon emission computed tomography are conventional imaging procedures for evaluating cellular metabolism and molecular markers using specific radiotracers. PET has become an indispensable procedure for the initial assessment and post-therapy evaluation in clinical oncology. PET can be used to evaluate the biological processes associated with disease progression and the therapeutic response at the cellular and molecular levels. Optical imaging using fluorescence and bioluminescence is used in preclinical studies; however, its clinical applications are limited owing to the limited penetration of these signals in the tissue. Magnetic resonance imaging (MRI) is widely used in clinical examination with contrast materials, such as gadolinium-based agents, superparamagnetic iron oxide, and perfluorocarbon labeled with fluorine-19; however, these materials are non-specific. Therefore, nuclear medicine imaging is a standard procedure for evaluating anticancer immune responses as well as viability of cancer cells [1]. In the case of immunotherapy and cell-based therapy, tracking of particular cell subsets using a relevant radiopharmaceutical that binds to specific cells may provide insight, leading to the understanding of the role of immune cells and to optimizing the efficacy of cancer therapy [2,3,4]. Molecular imaging may be promising for evaluating the therapy response and provide useful information to increase the benefit of anti-cancer therapy. 

Conventional response evaluation criteria rely on morphological parameters; on the other hand, metabolic parameters obtained from 2-deoxy-2-[^18^F] fluoro-D-glucose (FDG)-PET are the other representative surrogate markers. The histological response to anticancer therapy depends on the mode of action of therapeutic modalities. The tumor response to specific molecule-targeting drugs and immune checkpoint inhibitors (ICIs) is different from conventional chemotherapy in terms of temporal metabolic alteration and morphological changes after the therapy. FDG accumulation reflects the glucose metabolism of cancer cells as well as immune cells in the tumor, which differs among patients according to their function; however, FDG-PET can evaluate the viability of an entire tumor. Treatment-induced metabolic changes serve as an early indicator of therapy effectiveness and prognosis. The current approaches to anticancer therapy target the tumor microenvironment as well as anti-tumor immunity. Immunotherapy shows the distinctive phenomenon of immune-related tumor responses. Accumulated data of immune-related response patterns have led to the modification of the conventional response criteria. The role of glucose metabolism in lymphoid tissue has attracted attention as an imaging biomarker for the prognosis of patients after immunotherapy. On the other hand, specific imaging and tracking of cancer cells or immunological cell subsets can elucidate therapy response in complex interactions of these cells in the tumor microenvironment.

In this review, we discuss briefly the novel anticancer therapies targeting the tumor microenvironment, focusing on tumor immunity and metabolism, and the role of imaging as a biomarker of therapy-related immunological mechanisms. Considering tumor heterogeneity and individual variations in therapy response, a radiomics approach with quantitative features of multimodal images and deep learning algorithm with reference to pathologic and genetic data has the potential to improve response assessment for emerging cancer therapies. Molecular imaging in the therapy monitoring plays a definitive role in personalized therapeutic strategies within the framework of precision medicine.

## 2. Glucose Metabolism in Cancer Cells 

All cells basically require nutrients to generate energy for cell proliferation, differentiation, and biosynthesis of macromolecules. Glucose uptake and metabolism are significantly elevated in cancer cells [5]. PET studies have revealed that a high glycolytic rate and pyruvate oxidation in the mitochondria are correlated with cell proliferation. Altered glucose metabolisms, including the metabolic switch from aerobic to anaerobic glycolysis, are known as the Warburg effect [6,7]. The Warburg effect has been simply understood as stated above for many decades; however, a breakthrough of its underlying mechanism has been made recently to explain the Warburg effect in the context of cancer metabolism [8,9,10,11]. One of the most important factors to account for the biological aggressiveness and resistance of tumor to therapy is regarded as tumor hypoxia [11,12]. Accelerated proliferation and metabolism of cancer cells lead to an imbalance of oxygen demand and insufficient oxygen supply in many solid tumors [13,14]. Antineoplastic drugs and ionizing radiation cause oxidative stress through reactive oxygen species (ROS) generation in cancer cells, which results in apoptosis. However, cancer cells can survive in a hypoxic area, which is within 100 μm from tumor vessels, suppressing ROS synthesis, where the transcription factor, hypoxia inducible factor-1 (HIF-1), is activated in response to the hypoxia [15]. 

Various genes responsible for adaptation to hypoxic metabolism from oxidative phosphorylation to glycolytic ATP production are induced [16,17]. The Warburg effect accounts for the invasion and metastases of cancer cells through the formation of premetabolic niche and epithelial mesenchymal transition (EMT), increased erythropoiesis through erythropoietin upregulation, and reoxygenation of the hypoxic area through angiogenesis to escape from hypoxia [18,19]. Production of glucose transporter 1 and glycolytic enzymes is induced by an α-subunit of HIF-1 (HIF-1α) to enhance glucose uptake and anaerobic glycolysis [20,21]. Tumor aggressiveness and resistance are evaluated by FDG-PET on the basis of enhanced glucose metabolism that may be a possible therapeutic efficacy marker.

## 3. Therapy Monitoring with Imaging Biomarkers of FDG-PET

FDG-PET is currently the standard procedure for response evaluation of cancers, owing to its availability and standardization. The therapy responses of cancer and immune cells are diverse according to the therapeutic modalities and mode of action of drugs. Therefore, individualized evaluation criteria based on therapeutic agents should be appropriate. The therapeutic regimen and time from administration, immune function, temporal changes in size and attenuation of tumor on imaging, and proliferation, invasion, differentiation, vascularity, and interstitial findings on pathological examination are useful clinical information that supports response evaluation. 

PET is utilized for therapy monitoring of tumors based on the quantitative information of tracer uptake. The quantitative value depends on the tracer uptake and retention of the tracers, which vary greatly depending on the biochemical properties of tracers and the intracellular metabolism of cancer cells [22]. FDG is a representative PET tracer utilized for these purposes. FDG uptake depends on the cellular metabolism of glucose characterized by the expression of glucose transporters and glycolytic enzymes. Temporal changes in the uptake after therapy vary greatly depending on the mode of therapeutic actions. A fundamental quantitative index is the standardized uptake value (SUV), defined as the ratio of the radioactivity concentration in a defined region (MBq/mL) to the injected radioactivity that is corrected for total body mass (MBq/g). The maximum SUV (SUV_max_) is the value of a single voxel used to evaluate tumor viability, aggressiveness, and prognosis of the tumor [23,24,25]. 

SUV_peak_ is another index determined by averaging the tracer uptake in the region of interest within the tumor to maximize the value. Therefore, SUV_peak_ is less susceptible to partial volume effects than SUV_max_. Because the amount of FDG accumulated differs between white adipose tissue and other normal tissues such as muscle in the fasting state, the normalized SUV using the lean body mass (SUV_lean_, SUL) will, therefore, be appropriate for comparing SUV between obese patients and lean patients [26].

Tumor size has been essential for response evaluation. Total lesion glycolysis (TLG) and metabolic tumor volume (MTV) are more complicated parameters that reflect both tracer uptake and tumor volume, and they have been recognized as useful indictors for response evaluation [27,28]. A recent meta-analysis has suggested that TLG and MTV are better predictors for evaluating treatment outcomes than SUVs in patients with lung cancer [29].

The response of solid tumors to chemotherapy and radiotherapy is evaluated on the basis of post-therapeutic changes in the unidimensional largest diameter assessed by imaging methods, such as computed tomography (CT) (Table 1) [30]. Cellular function and metabolism-based response evaluation criteria based on PET/CT have been shown to be relevant for patients undergoing molecular-targeted therapy as well as chemotherapy. There have been two representative criteria available for response evaluation: European Organization for Research and Treatment of Cancer (EORTC) criteria and PET Response Criteria in Solid Tumors (PERCIST) (Table 1) [31,32].

PET/CT-based response evaluation varies according to the therapeutic drugs used, such as conventional chemotherapeutic agents, molecular targeted drugs, and immune checkpoint inhibitors, because glucose metabolism depends on the metabolic alteration of glucose after these treatments. Metabolic changes usually occur ahead of volume reduction in molecular targeted therapy. A preliminary study suggests the usefulness of FDG-PET for the early prediction of gefitinib efficacy based on the 60% decrease in glucose metabolism as early as two days after treatment initiation in patients with non-small cell lung cancer (NSCLC) [36]. This and subsequent data facilitated the cognizance of the need to establish relevant response evaluation criteria [37].

ICIs are epoch-making therapeutics for many types of cancer through the host immune system to eradicate malignant cells within the acceptable range of toxicity. ICIs include antibodies against cytotoxic T-lymphocyte-associated protein 4 (CTLA-4) and programmed cell death receptor 1 (PD-1) and its ligand (PD-L1). Although these therapies are effective even for chemotherapy-resistant tumors, an established biomarker to predict therapeutic efficacy is as yet not determined, because the interaction between tumor cells and host anti-tumor immunity may be too complicated to be evaluable by a single metabolic parameter [22]. However, a recent study has indicated that FDG uptake is an independent prognostic factor for nivolumab therapy, although PD-L1 expression or plasma nivolumab concentration is not a predictor of early therapeutic efficacy [38]. FDG-PET seems to be useful for monitoring early response to an anti-PD-1 antibody. 

## 4. Relationship between PD-L1 Expression and Monitoring of Immunotherapy by FDG-PET

Several studies have shown the positive correlation of PD-L1 expression with FDG accumulation in patients with different types of human cancers [39,40,41,42,43,44,45,46,47,48,49,50]. The relationship between PD-L1 expression and FDG uptake based on previous reports is listed in Table 2. Seven of eleven studies focused on lung cancer, and four were on cancers originating from the colon, rectum, bladder, breast, and nasopharynx. A significant correlation of FDG uptake with the expression of PD-L1 by immunohistochemistry was also observed in small cell lung cancer (SCLC) (Table 2). However, two studies described that the expression level of PD-L1 was not significantly linked to the count of tumor infiltrative lymphocytes (TILs), such as CD4, CD8, and Foxp3-regulatory T cells (Tregs) in NSCLC [37,38]. On the other hand, three studies indicated that there was a significant association between SUV_max_ and TIL count in patients with SCLC, NSCLC, and breast cancer [41,44,46]. On the basis of these lines of evidence, the relationship between PD-L1 expression and FDG uptake appears not significant, whereas the correlation of TILs with FDG accumulation appears to vary depending on cancer type or histology. Further investigations are necessary to elucidate the relationship between FDG uptake and tumor microenvironment in human neoplasms. 

It remains unclear whether FDG PET can successfully differentiate responders from non-responders at an early phase after the initiation of treatment with ICIs. Recently, there have been several reports on the usefulness of FDG PET for the monitoring of the therapeutic efficacy of ICIs in patients with malignant melanoma and NSCLC. One preliminary study suggests that FDG PET can detect complete responders to PD-1 blockade drugs at 2 weeks after the initiation of treatment for patients with advanced melanoma [51]. Annovazzi et al. reported that FDG PET at 3 to 4 months after ICI treatment can accurately show the response to treatment and predict long-term survival in 57 patients with advanced melanoma [52]. In a recent retrospective study of 104 patients with advanced melanoma treated with PD-1 blockade drugs, most of the patients with partial response determined on the basis of Response Evaluation Criteria in Solid Tumors (RECIST) achieved complete metabolic response (CMR) as shown by FDG PET at 1 year after the initiation of PD-1 blockade drugs, and most of these patients with CMR at 1 year later revealed continued response to treatment thereafter [53]. These findings suggest the therapeutic significance of FDG PET for predicting long-term survival. In patients with NSCLC, FDG PET is also reported to be useful as a predictive marker of early response after PD-1 blockade drug initiation [34]. For the assessment of FDG accumulation, TLG and MTV were found to be better for evaluating metabolic activity than SUV_max_ based on FDG uptake [34]. Other studies also indicated the clinical usefulness of FDG PET for evaluating the early response to PD-1 blockade drugs in patients with advanced NSCLC [54,55,56]. Moreover, several studies exhibited the prognostic significance of FDG uptake prior to PD-1 blockade drugs [57,58,59]. 

## 5. Limitations and Prospects of Response Evaluation of ICI Therapy by PET

Limitations of FDG-PET for response evaluation in anti-neoplastic drugs, radiotherapy, and immunotherapy have been raised [22]. FDG shows high accumulation in inflammatory foci owing to the enhanced glycolysis in inflammatory cells, such as activated T cells, macrophages, and neutrophils, resulting in a false-positive assessment despite a good therapeutic response. Post-therapeutic biologic mechanisms after immune checkpoint inhibition represent unique response phenomena in tumor, such as an initial enlargement caused by the infiltration of inflammatory immune cells into the tumor called pseudoprogression, followed by the decrease in both the size and rate of glucose metabolism [60,61,62]. On the basis of these findings, RECIST has been modified to define the current immune-related response evaluation criteria; immune-related RECIST (irRECIST) and immune RECIST (iRECIST) (Table 1) [63,64]. The clinical significance of these criteria should be validated by prospective clinical trials, because even a growing body of study has so far not clarified the frequency and temporal changes in the pseudoprogression. 

The prediction of response to ICIs is important in terms of avoiding unnecessary toxicities in responders and introducing another potent treatment option in non-responders. Assessment of tumor response to ICIs by the morphology-based criteria as above seems insufficient in terms of pseudoprogression and delayed tumor shrinkage, although the frequency and significance of these phenomena for response assessment are undetermined. FDG-PET reflects the increased rate of glucose consumption in a broad spectrum of proliferating and biologically active cells. This is a limitation for evaluating tumor viability on the one hand, but on the other hand, FDG-PET is capable of evaluating anti-tumor immune response and resulting tumor resolution. A retrospective study has suggested immunotherapy-modified PERCIST (imPERCIST) by changing the definition of PMD determined not by the appearance of new lesions, but by an increase in the sum of peak SUL (SUL_peak_) [65]. 

Monitoring the glucose metabolism in lymphoid tissues such as the spleen can be used to predict immune response, because the spleen plays pivotal roles in the recruitment and activation of immune cells in the tumor microenvironment, especially in patients treated with ICIs. For example, an increased rate of spleen glucose metabolism simply evaluated with SUV_max_ or spleen-to-liver ratio of SUV_mean_ has been shown to indicate a poor prognosis in patients treated for a wide range of malignant tumors [63,64,65]. However, inconsistent results have been obtained [66,67]; therefore, prospective studies are necessary to determine the correlation of spleen glucose metabolism with anticancer therapies including radiotherapy. The criteria for response evaluation by FDG-PET must be specifically revised to be consistent with the temporal alteration of glucose metabolism in accordance with various anti-cancer therapies. Malignant lymphoma is a representative malignancy benefited by the response evaluation with FDG-PET. The role of FDG-PET has been reported in evaluating early and interim treatment responses to ICIs in patients with lymphoma [68].

FDG-PET plays an important clinical role for these purposes. Since glucose metabolism reflects not only the viability of cancer cells, but also all other cells involved in immune reactions in the tumor microenvironment, an enhanced uptake of FDG does not completely indicate tumor progression. The pseudoprogression phenomenon and enhanced anaerobic glycolysis followed by the therapy-induced hypoxia are not uncommon in the anticancer therapy [57]. Neoplastic-cell-specific imaging agents have potential use of evaluating residual cancer cells; however, phenotypic changes due to genetic mutation after therapy may decrease the efficiency of such specific agents. Amino-acid PET tracers are currently used to evaluate therapeutic efficacy; however, metabolic diversity and instability acquired during cancer progression after therapy are possible sources of inaccuracy.

Cancer-specific radiotracers can be used to evaluate tumor viability and therapeutic effect more accurately, regardless of the temporal immune response and residual interstitial tissue after complete eradication of tumor cells. Moreover, immune checkpoint molecule-specific radiolabeled tracers, such as antibodies against PD-1, PD-L1, and CTLA-4, have been examined for visualizing key molecules of immune-checkpoint pathways and immune responses [69,70,71]. Engineered antibody-based PD-L1 antagonists conjugated with ^64^Cu-DOTA and CD8^+^ T cell-targeted peptides labeled with ^68^Ga-NOTA have demonstrated favorable tumor-to-background ratios and the uptake reflecting tumor response to anti-PD-1 and anti-CTLA-4 therapies in mouse xenograft models, respectively [72,73,74].

## 6. Cancer Metabolomics as Target of Therapy and Response Evaluation by PET

Tissue homeostasis involving cellular metabolism and function of both stromal and immune cells comprises tumor ecosystem that is a critical determinant of the viability of cancer cells and cancer-responding immune cells. In relation to the disruption of homeostasis in tumor microenvironments characterized by acidic, hypoxic, or depleted critical nutrients, such microenvironments are regarded as novel targets of cancer therapy [75,76]. 

Conventional chemotherapeutic agents targeting cancer metabolism are 5-fluorouracil and gemcitabine. On the other hand, several clinical trials with amino acid metabolism-targeted therapy using l-asparaginase have been performed for acute lymphoblastic leukemia (ALL) [77]. The specificity of metabolic preferences in the tumor was considered to provide possibilities of therapy; however, the deprivation of at least two major nutrients, glucose and glutamine, has been shown to unsuccessfully eliminate cancer cells or induce antineoplastic immune cells owing to the metabolic cooperation and competition between them within the tumor microenvironment. Previous studies have shown that the metabolic phenotype is not specific to cancer cells but reflects the biological features of proliferating cells, including immune cells [78,79]. 

Many types of radiopharmaceuticals, such as ^18^F-labeled amino acids, have been evaluated for targeting neoplastic cells as candidates for the specific imaging of malignant tumors [80,81,82]. One example is 3-[^18^F] fluoro-l-α-methyltyrosine (^18^F-FAMT) [83,84], which is transported by l-type amino acid transporter 1 (LAT-1) specifically expressed on various cancer cells [85]. The clinical usefulness of ^18^F-FAMT PET for evaluating prognosis and therapeutic response has been reported [86,87]. The uptake of ^18^F-FAMT has been shown to correlate with PD-L1 expression in patients with advanced NSCLC [88]. Therefore, inhibition of LAT1 is a possible anti-cancer therapy for a wide range of malignant tumors [89]. Following clinical trials of LAT1-targeted therapy, FDA has approved JPH203 as orphan drug destination for the treatment of biliary tract cancer in 2022 [90]. Besides glucose, glutamine is another material that contributes to metabolic fuel and is the primary nitrogen source for DNA replication in various cancer cells [91]. Asparagine promotes cell proliferation and survival in the absence of glutamine and its biosynthesis requires glutamate and nitrogen [92]. Therefore, l-asparaginase induces apoptosis of ALL cells through the reduction in blood asparagine levels as mentioned above [77,93]. A recent study showed that a glutamine antagonist induces tumor regression in mice by suppressing the metabolism of both glutamine and glucose, resulting in a microenvironment favorable for T-cell effector function [94,95].

Cancer cells can use energy sources by autophagic degradation of macromolecules; therefore, starving strategies, such as deprivation of amino acids, are unlikely to be successful for cancer therapy [96]. In addition, cellular metabolism depends on cell lineage, and the tumor microenvironment can significantly affect cellular metabolism [97]. Recent studies have shown the function of autophagy as non-apoptotic, the so-called autophagic cell death, and have led to the conventional understanding of the resistance mechanism against stress environments, such as hypoxia, chemotherapy, radiotherapy, and starvation [18]. Therefore, regulation of autophagy is considered a novel treatment strategy for malignant tumors [98]. Clinical trials have been conducted using phosphoinositide-3-kinase inhibitors and lysosomal inhibitors, such as hydroxychloroquine [99,100]. One of the recent noticeable studies on autophagy-targeted therapy uses a specific inhibitor for autophagosome formation that is a key step for following lysosomal degradation in the autophagy-lysosome system [101].

## 7. Possible Role of PET with Radiomics and Artificial Intelligence for Response Evaluation

Efficient biomarkers are expected for the purpose of determining therapeutic indication and correlating with outcome, because novel anticancer therapies are not mainly organ-oriented nor pathological entities, but they target a specific gene mutation or metabolic phenotype. Molecular imaging is, therefore, a potential surrogate marker that can non-invasively evaluate disease status and therapy outcomes. 

Radiomics is a quantitative analysis to correlate large-scale imaging features to biological and clinical endpoints [102]. The quality of diagnosis, prognostic stratification and treatment response will be increased by radiomics procedure with complimentary information from clinical data, treatment response, and genetic assays. The intratumoral heterogeneity is one of the main targets measured by radiomic procedure that combines objective image features, called “radiomic features”, including size, shape, intensity, and texture with a machine learning algorithm to diagnose the pathophysiology of various diseases. Recent advancements in machine learning-based methods have improved diagnosis, staging, and response assessment for personalized therapy in oncology. Associations between radiomic phenotype and gene expression were found in various types of cancer [103,104,105]. The establishment of accurate prediction models for treatment response to ICIs is an important issue in the clinical practice. A well-known AI product of PLUS.LUNG.NODULE (Plusman LLC, Tokyo, Japan) has been used to automatically extract tumors and evaluate their attributes and features. Machine learning is applied to estimate the magnitudes of effect using clinical information, treatment data, as well as image data as inputs, and combine existing biomarkers to establish a model that measures the potential efficacy of each treatment and selects the optimal treatment for the designated patient. For example, a combination of CT imaging parameters and clinical features can provide therapeutic benefits by identifying genotypic information about anaplastic lymphoma kinase (ALK) for deciding the use of crizotinib for therapy in patients with lung adenocarcinoma [106]. 

Radiomic assessment is formerly performed mainly with CT and MRI; however, radiomics with PET will be in use not only for initial staging and prognostic stratification, but also post-therapy assessment owing to the merit of evaluating broad spectrum of tissue characterization from vast amount of tissue metabolism to specific cell targeting in the tumor microenvironment and the relatively high contrast between signal and background [107,108]. Studies have shown the relevant role of a PET radiomics-based biomarker for the prediction of therapeutic response to various therapeutic options as compared with conventional biopsy-based assays that may not always represent the relevant pathology of a heterogeneous tumor [109,110,111]. Common PET radiomics procedures for response evaluation of NSCLC have been employing volume and metabolism of the tumor and sometimes the muscle, using quantitative indices such as SUV, which are combined with PD-L1 immunohistochemical staining and driver mutations of the tumor, patient characteristics such as smoking history, age, sex, ECOG Performance Status, and blood examination to generate a feature map as a test set for further validation. Artificial intelligence can integrate this information with radiomics analysis for more efficient evaluation. Therapeutic decision-making on the basis of response and toxicity is critical for novel therapeutic modalities; therefore, precise predictors are required for every treatment. A multicohort study in patients with advanced solid tumors has shown the usefulness of a radiomic signature of CD8-positive cells for inferring clinical outcomes of patients by immunotherapy using anti-PD-1 or anti-PD-L1 antibodies [112]. A first-in-human study of CD8-targeted PET imaging demonstrated increased ^89^Zr-labeled anti-CD8 minibody accumulation in tumors and CD8-rich tissues such as spleen, bone marrow, and lymph nodes, which correlated with response to immunotherapy [113]. 

There have been numerous works reporting the use of response monitoring by radiomics assessment with PET/CT in patients with various types of malignancies, such as lung, esophagus, and colon cancers and malignant lymphoma [105,111,114,115,116]. Although many studies have supported the utility of PET radiomics, concerns about the robustness and reproducibility of the results have been raised, because most of the works have been small, retrospective, and monocentric cohorts without external validation [117]. Over the last few years, studies have increased quality and reliability with larger cohorts and robust statistical analysis on the basis of a number of publications and recommendation initiatives to improve standardization and reproducibility [118,119,120]. Technical improvement including data acquisition, tumor segmentation, feature extraction, and modeling strategies and rapidly developing deep learning technology has been proposed [121,122]. Although radiomics has drastically progressed over the past decade, its standardization is required to be established as a therapy monitoring procedure based on large-cohort prospective clinical trials [108,123].

## 8. Conclusions

Recent progress in cancer immunotherapy has promised the need for establishing effective biomarkers for therapy monitoring. A well-established FDG-PET seems to be a useful surrogate marker, because it reflects increased glucose consumption in a broad spectrum of biologically and immunologically active cells in the tumor microenvironment. Considering future therapeutic targets including metabolomics and autophagy, appropriate evaluation will benefit from radiomics approach that combines objective image features with a machine learning algorithm as well as pathologic and genetic information on the basis of tumor heterogeneity and individual variation in therapy response. Optimizing molecular imaging will strengthen the clinical role for monitoring therapy within the framework of precision medicine.

## Figures and Tables

**Table 1:** Conventional/immune-related response evaluation criteria and response evaluation criteria using FDG-PET for solid tumors.

Criteria	Measurement	CMR/CR	PMR/PR	PMD/PD	Reference
RECIST 1.1	Unidimensional (LD for non-nodal lesions; LPD for LN)	Disappearance of all target lesions < 10 mm for any pathological LN	≥30% reduction	≥20% and ≥5 mm increase, new lesion, or non-target PD	[30]
irRECIST	Unidimensional (LD for non-nodal lesions; LPD for LN)	Disappearance of all target lesions	≥30% reduction	≥20% and ≥5 mm increase, or non-target PD	[33]
iRECIST	Unidimensional (LD for non-nodal lesions; LPD for LN)	Disappearance of all target lesions	≥ 30% reduction	≥20% and ≥5 mm increase, or non-target PD, new lesion confirmed at the next assessment	[34]
EORTC	SUV_max_	Complete resolution of FDG uptake in all lesions	>25% reduction in the sum of SUV_max_ after more than one cycle of treatment	>25% increase in the sum of SUV_max_ or appearance of new lesions	[31]
PERCIST	SUL_peak_	Complete resolution of FDG uptake in all lesions	≥30% reduction of SUL_peak_ and an absolute drop of 0.8 SUL_peak_ units	>30% increase in SUL_peak_ and an absolute increase of 0.8 SUL_peak_, or appearance of new lesions	[32]
imPERCIST	SUL_peak_	Complete resolution of FDG uptake in all lesions	≥30% reduction of SUL_peak_ and an absolute drop of 0.8 SUL_peak_ units	>30% increase in SUL_peak_ and an absolute increase of 0.8 SUL_peak_, or new lesions included in the sum of SUL_peak_	[35]

FDG-PET, 2-deoxy-2-[^18^F] fluoro-D-glucose positron emission tomography; RECIST, response evaluation criteria in solid tumors; irRECIST, immune-related RECIST; iRECIST, immune RECIST; EORTC, the European Organization for Research and Treatment of Cancer; PERCIST, PET Response Criteria in Solid Tumors; imPERCIST, immunotherapy-modified PERCIST; CMR/CR, complete metabolic response/complete response; PMR/PR, partial metabolic response/partial response; PMD/PD, progressive metabolic disease/progressive disease; LD, largest diameter; LPD, largest perpendicular diameter; LN, lymph nodes; SUV_max_, maximum standardized uptake value; SUL_peak_, peak lean body mass standardized uptake value.

**Table 2:** Relationship among PD-L1 expression, TILs, and FDG uptake in various cancer types.

Cancer Type	Histology	No. of Patients	Correlation between FDG Uptake and PD-L1 Expression *p*-Value (PD-L1 Clone)	Correlation between FDG Uptake and TILs	Reference
Lung cancer	SCC/AC/other	579	<0.001 (SP142)	NA	[38]
Lung cancer	SCC	167	0.02 (E1L3N)	Not significant	[39]
Lung cancer	AC	315	0.01 (E1L3N/38-8)	Not significant	[40]
Lung cancer	SCC	84	0.035 (28-8)	NA	[41]
Bladder cancer	UC/SCC/SRC	63	0.032 (NA)	NA	[42]
Lung cancer	SCLC	98	0.36 (E1L3N)	Significant	[43]
Lung cancer	SCC/AC	362	0.001 (28-1)	NA	[44]
Colon cancer	AC	65	0.001 (28-8)	NA	[45]
Lung cancer	SCC/AC	122	0.012 (NA)	Significant	[46]
NPC	SCC	84	<0.001 (SP263)	NA	[47]
OSCC	SCC	59	0.003 (28/8)	Not significant	[48]
Breast cancer	AC	97	<0.001 (28-8)	Significant	[49]

PD-L1, programmed death ligand-1; FDG, 2-deoxy-2-[^18^F] fluoro-D-glucose; TILs, tumor infiltrative lymphocytes; SCC, squamous cell carcinoma; AC, adenocarcinoma; SCLC, small cell lung cancer; UC, urothelial cancer; SRC, signet ring cell carcinoma; NPC, nasopharyngeal carcinoma; OSCC, oral squamous cell carcinoma; NA, not applicable.

## Data Availability

Not applicable.

## Abbreviations

PET: Positron emission tomography; FDG: 2-deoxy-2-[^18^F] fluoro-D-glucose; ICIs: immune checkpoint inhibitors; AI: Artificial intelligence; ROS: reactive oxygen species; HIF-1: hypoxia inducible factor-1; EMT: epithelial mesenchymal transition; SUV: standardized uptake value; SUV_max_: maximum SUV; SUV_peak_: peak SUV; SUV_lean_, SUL: normalized SUV using the lean body mass; SUL_peak_: peak SUL; TLG: Total lesion glycolysis; MTV: metabolic tumor volume; CT: computed tomography; EORTC: European Organization for Research and Treatment of Cancer; PERCIST: PET Response Criteria in Solid Tumors; NSCLC: non-small cell lung cancer; CTLA-4: cytotoxic T-lymphocyte-associated protein 4; PD-1: programmed cell death receptor-1; PD-L1: programmed cell death receptor-ligand 1; SCLC: small cell lung cancer; TILs: tumor infiltrative lymphocytes; Tregs: Foxp3-regulatory T cells; RECIST: Response Evaluation Criteria in Solid Tumors; CMR: complete metabolic response; irRECIST: immune-related RECIST; iRECIST: immune RECIST; imPERCIST: immunotherapy-modified PERCIST; ALL: acute lymphoblastic leukemia; ^18^F-FAMT: 3-[^18^F] fluoro-l-α-methyltyrosine; LAT-1: l-type amino acid transporter 1; ALK: anaplastic lymphoma kinase; MRI: magnetic resonance imaging.
